# Supplementary material for: Common virulence gene expression in adult first-time infected malaria patients and severe cases
Source: eLife. 2021 Apr 28;10:e69040. doi: 10.7554/eLife.69040 (PMC8102065; doi:10.7554/eLife.69040)
Supplement: Transparent reporting form [file elife-69040-transrepform.docx]

***eLife’s* transparent reporting form**

We encourage authors to provide detailed information *within their submission* to facilitate the interpretation and replication of experiments. Authors can upload supporting documentation to indicate the use of appropriate reporting guidelines for health-related research (see [EQUATOR Network](http://www.equator-network.org/%20)), life science research (see the [BioSharing Information Resource](https://biosharing.org/)), or the [ARRIVE guidelines](http://www.plosbiology.org/article/info:doi/10.1371/journal.pbio.1000412) for reporting work involving animal research. Where applicable, authors should refer to any relevant reporting standards documents in this form.

If you have any questions, please consult our Journal Policies and/or contact us: [editorial@elifesciences.org](mailto:editorial@elifesciences.org).

**Sample-size estimation**

- You should state whether an appropriate sample size was computed when the study was being designed
- You should state the statistical method of sample size computation and any required assumptions
- If no explicit power analysis was used, you should describe how you decided what sample (replicate) size (number) to use

Please outline where this information can be found within the submission (e.g., sections or figure legends), or explain why this information doesn’t apply to your submission:

No explicit power analysis was used since the analysis is exploratory nature. The number of samples available highly depends on the occurrence of travelers returning from Africa with certified *P. falciparum* infection and substantial parasitemia for analysis. Since we sampled for over 5 years, we decided to stop further sampling at the end of 2018 to finalize the study.

**Replicates**

- You should report how often each experiment was performed
- You should include a definition of biological versus technical replication
- The data obtained should be provided and sufficient information should be provided to indicate the number of independent biological and/or technical replicates
- If you encountered any outliers, you should describe how these were handled
- Criteria for exclusion/inclusion of data should be clearly stated
- High-throughput sequence data should be uploaded before submission, with a private link for reviewers provided (these are available from both GEO and ArrayExpress)

Please outline where this information can be found within the submission (e.g., sections or figure legends), or explain why this information doesn’t apply to your submission:

A single, unique, ex vivo RNA and a plasma sample was obtained from individual patients (n=32). Each patient was assigned to either (i) first-time infected or pre-exposed and (ii) severe or non-severe malaria groups according to their serological data or WHO criteria. Within each group a single patient sample represents a biological replicate. Serological assays were done in technical replicates, where samples were measured in duplicates or triplicates.

Outliers, inclusion/exclusion criteria and how outliers were handled are described in the manuscript in details, e.g. both RNA samples with lower *P. falciparum* reads were not included in the de novo assembly, but in the differential expression analysis.

The RNAseq data were uploaded to NCBI (BioProject ID: PRJNA679547).

**Statistical reporting**

- Statistical analysis methods should be described and justified
- Raw data should be presented in figures whenever informative to do so (typically when N per group is less than 10)
- For each experiment, you should identify the statistical tests used, exact values of N, definitions of center, methods of multiple test correction, and dispersion and precision measures (e.g., mean, median, SD, SEM, confidence intervals; and, for the major substantive results, a measure of effect size (e.g., Pearson's r, Cohen's d)
- Report exact p-values wherever possible alongside the summary statistics and 95% confidence intervals. These should be reported for all key questions and not only when the p-value is less than 0.05.

Please outline where this information can be found within the submission (e.g., sections or figure legends), or explain why this information doesn’t apply to your submission:

All statistical methods are explained and justified in the Methods section. Furthermore, details about the presented measures are given in the figure legends.

(For large datasets, or papers with a very large number of statistical tests, you may upload a single table file with tests, Ns, etc., with reference to sections in the manuscript.)

**Group allocation**

- Indicate how samples were allocated into experimental groups (in the case of clinical studies, please specify allocation to treatment method); if randomization was used, please also state if restricted randomization was applied
- Indicate if masking was used during group allocation, data collection and/or data analysis

Please outline where this information can be found within the submission (e.g., sections or figure legends), or explain why this information doesn’t apply to your submission:

Since this is an operational study, patients were not allocated into study groups. Individual serological reactivities and clinical diagnoses were used to allocated patients to the respective study groups. This is described in the beginning of the Results section in the paragraph ‘cohort characterization’.

**Additional data files (“source data”)**

- We encourage you to upload relevant additional data files, such as numerical data that are represented as a graph in a figure, or as a summary table
- Where provided, these should be in the most useful format, and they can be uploaded as “Source data” files linked to a main figure or table
- Include model definition files including the full list of parameters used
- Include code used for data analysis (e.g., R, MatLab)
- Avoid stating that data files are “available upon request”

Please indicate the figures or tables for which source data files have been provided:

We provide all relevant raw data from serological assays (supplement file 1), expression analyses (supplement file 3–5, 8–11) and sequences from assembled contigs (source data 1) in the supplement.
